# Retraction: Up-Regulation of Sonic Hedgehog Contributes to TGF-β1-Induced Epithelial to Mesenchymal Transition in NSCLC Cells

**DOI:** 10.1371/journal.pone.0205290

**Published:** 2018-10-02

**Authors:** The PLOS ONE Editors

After publication of this article [1], concerns were raised about Figs 1, 3, 5, 7:

- It was raised that the GAPDH data shown in Fig 1C are similar to the β-actin data in Fig 3D, although the aspect ratios and brightness/contrast levels differ in the two figures.
- Horizontal and vertical discontinuities were noted in Fig 7A between the two lanes and between the Shh and β-actin bands.
- Concerns were raised about vertical discontinuities between the first and second lanes in the upper panels of Figs 1C, 3D, and 5D. For these figures, it was further raised that the loading control and experimental protein blots do not align and appear to have been derived from different gels/blots based on band separation in the corresponding figure panels.

Wayne State University investigated this work and found evidence of image falsification and manipulation. The original data supporting the results in question were not provided or found during the investigation.

In light of these concerns, and in line with the institution’s recommendation, the *PLOS ONE* Editors retract this article.

MYM, SA, AA, SG and FHS did not respond to the notification for retraction.
